# *Mediterraneibacter catenae* SW178 sp. nov., an intestinal bacterium of feral chicken

**DOI:** 10.7717/peerj.11050

**Published:** 2021-04-27

**Authors:** Supapit Wongkuna, Sudeep Ghimire, Surang Chankhamhaengdecha, Tavan Janvilisri, Joy Scaria

**Affiliations:** 1 Department of Biochemistry, Faculty of Science, Mahidol University, Bangkok, Thailand; 2 Department of Veterinary and Biomedical Sciences, South Dakota State University, Brookings, South Dakota, United States; 3 South Dakota Center for Biologics Research and Commercialization, Brookings, South Dakota, United States; 4 Department of Biology, Faculty of Science, Mahidol University, Bangkok, Thailand

**Keywords:** Mediterraneibacter catenae, Gut microbiome, Culturomics, Feral chicken, Taxonogenomic, Novel species

## Abstract

A Gram-positive, coccobacillus, white raised and circular with an entire edge colony, and obligately anaerobic bacterium, strain SW178 was isolated from the cecum content of feral chickens in Brookings, South Dakota, USA. The most closely related strain based on 16S rRNA gene sequence analysis of strain SW178 was *Mediterraneibacter torques* ATCC 27756^T^ (*Ruminococcus torques* ATCC 27756^T^) with 96.94% similarity. The genome of strain SW178 is 3.18 Mbp with G+C content of 46.9 mol%. The optimal temperature and pH for growth in modified brain heart infusion (BHI-M) medium were 45 °C and pH 7.5, respectively. The sole carbon sources of the strain were dextrin, L-fucose, D-galacturonic, α-D-glucose, L-rhamnose and D-sorbitol. The primary cellular fatty acids were C_14 : 0_, C_16 : 0_ and C_16 : 0_ dimethyl acetal (DMA). Based on the genotypic and phenotypic comparison, we proposed that strain SW178 belong to the genus *Mediterraneibacter* in the family *Lachnospiraceae* as a novel species, in which the name *Mediterraneibacter catenae* is proposed. The type strain is SW178 (= DSM 109242^T^ = CCOS 1886^T^).

## Introduction

High diversity of bacterial community that inhabits the gastrointestinal tract of animals known as gut microbiota provides significant beneficial impacts on host health such as digestion of polysaccharides, development of immune system, and pathogen protection (Jandhyala et al., 2015; Thursby & Juge, 2017). In chicken, the gut harbors dense and complex microbial population that plays a part in poultry health and production. After colonization inside the gut, commensal bacteria can attach to the mucosa, forming a protective barrier that reduces the chance of pathogenic infections. These bacteria produce vitamins such as vitamins B and K, short chain fatty acids such as acetic acid, butyrate acid and propionic acid, which promote functions of the epithelial cells, including the construction of mucus layers, the development of immunity and the production of antimicrobial peptides. Conversely, the gut microbiota can carry pathogenic bacteria such as *Salmonella* and *Campylobacter*, in which their transmission and infection in humans contribute to a concerned issue in public health. The composition and function of the gut microbiota is influenced by multiple environmental factors, such as diet and medication. The most common taxa in the chicken gut include the genera *Enterococcus*, *Clostridium*, *Bacillus*, *Ruminococcus*, *Staphylococcus*, *Streptococcus* and *Lactobacillus*, belonging to the phylum Firmicutes. Furthermore, many members of other phyla such as Bacteroidetes, Proteobacteria and Actinobacteria can also be found in the gut of chicken (Gong et al., 2007; Lu et al., 2003). These bacteria distribute along regions of the gastrointestinal tract with different abundance. The cecum of chicken harbors the highest diversity and abundance of bacteria at 10^10^–10^11^ cells/g content, followed by the small intestine and crop with the concentration of 10^8^–10^9^ cells/g (Shang et al., 2018). In a balanced gut microbiota, commensal bacteria can control bacterial pathogens via competitive exclusion and host immune stimulation. For example, short chain fatty acids produced by gut microbiota have direct antimicrobial activity against enteric pathogens such as *Salmonella enterica* (Lamas et al., 2019). Therefore, modulation of the gut microbial community offers opportunities to improve host physiology.

Since poultry meat and eggs provide a major protein source in human nutrition worldwide, broiler chickens become the most common farmed animal. However, the expansion of poultry industry has revealed changes in the gut microbial community, which can have impacts on productivity and health of chickens. (Koenig et al., 2011; Qin et al., 2010). The alteration of microbial community is associated with several disorders and diseases. The disruption of normal gut microbiota facilitates the invasion of opportunistic pathogens that leads to infectious diseases contributing to negative impacts on chicken production (Dethlefsen et al., 2008). Although antibiotics have been used extensively in the poultry industry to reduce mortality from pathogen infection, they can lead to emergence of antibiotic-resistant bacteria. The administration of beneficial microbes to modify the gut microbiota becomes one of alternative strategies for maintaining chicken health. Probiotics drive health improvement through the variety of mechanisms associated with the modulation of host immunity and epithelial barrier function, the improvement of host metabolism, and the competition with harmful bacteria (Byndloss et al., 2017; Chen et al., 2012; Park et al., 2018). Therefore, isolation and cultivation known as the culturomics are crucial for discovery of unknown members of gut microbiota that may provide benefits to the host. (Ito et al., 2019; Rettedal, Gumpert & Sommer, 2014). Such demonstration can be used in development of probiotics to improve animal health, including clinical therapies. Furthermore, the culturomics facilitates biological investigation in interaction among bacterial species and between bacteria and host cells (Lagier et al., 2015; Sommer, 2015).

The phylum Firmicutes is a core composition of chicken gut microbiota. Many members of the family *Lachnospiraceae* have been isolated from chicken gut, except the genus *Mediterranneibacter*, which all typed species were discovered from human gut (Lagier et al., 2016; Rettedal, Gumpert & Sommer, 2014; Zou et al., 2019). The genus *Mediterraneibacter* was firstly described and named as *Ruminococcus*, in 1948 by Sijpesteijn (Sijpesteijn, 1951) before reclassification based on the genomic comparison by Togo in 2018 (Togo et al., 2018). *Ruminococcus* is phylogenetically heterogeneous, which was previously assigned into two separated clusters, Clostridium cluster XIVa and IV. The type species, *R. gauvreauii*, *R. lactaris* and *R. torques* are members of the cluster XIVa and are included in the family *Lachnospiraceae* (Domingo et al., 2008; Rainey, 2009a; Togo et al., 2018). Members of the cluster IV such as *R. flavefaciens*, *R. bomii* and *R. champanellensis* belong to the family *Ruminococcoceae* (Chassard et al., 2012; Moore, Cato & Holdeman, 1972; Rainey, 2009b). *Mediterraneibacter* species such as *M. faecis, M. glycyrrhizinilyticus, M. lactaris* and *M. torques* were later reclassified from *Ruminococcus* species in Clostridium cluster XIVa. These species are Gram-positive, coccoid or coccobacilli, non-motile obligately anaerobic bacteria belonging to the genus *Mediterraneibacter* of family *Lachnospiraceae* in the phylum Firmicutes, which dominate cecal microbiota of chicken (Jurburg et al., 2019; Oakley et al., 2014; Thomas et al., 2019). The G+C content of genome ranges from 42 to 45 mol%. The major metabolic products are acetic acid, formic acid and lactic acid. These phenotypic and genotypic characteristics imply biological differences from other members of the family *Lachnospiraceae*.

Here, we described phenotypic and genotypic characterization of strain SW178, isolated from the cecum of feral chickens as a new species. This strain has distinct characteristics compared to the closest valid species, *M. torques* ATCC 27756^T^ (Moore & Holdeman, 1974). We proposed *Mediterraneibacter catenae* sp. nov. strain SW178 (= DSM 109242^T^ = COS 1886^T^) as a novel member of the genus *Mediterraneibacter* in the family *Lachnospiraceae*.

## Materials & Methods

### Isolation and identification

Strain SW178 was isolated from the cecum of feral chicken in an anaerobic workstation (Coy Laboratory) containing 85% nitrogen, 10% hydrogen and 5 % carbon dioxide. Modified brain heart infusion (BHI-M) medium containing 37 g/l of BHI, 5 g/l of yeast extract, one ml of 1 mg/ml menadione, 0.3 g of L-cysteine, one ml of 0.25 mg/l of resazurin, one ml of 0.5 mg/ml hemin, 10 ml of vitamin and mineral mixture, 1.7 ml of 30 mM acetic acid, two ml of 8 mM propionic acid, two ml of 4 mM butyric acid, 100 µl of 1 mM isovaleric acid, and 1% of pectin and inulin, was used for strain isolation. To identify strain SW178, genomic DNA was extracted using a DNeasy Blood & Tissue kit (Qiagen, Hilden, Germany), according to the manufacturer’s instructions. Then 16S rRNA gene sequences were amplified using universal primer set 27F (5′- AGAGTTTGATCMTGGCTCAG-3′; Lane, 1991) and 1492R (5′- ACCTTGTTACGACTT- 3′; Stackebrandt & Liesack, 1993) (Lane, 1991; Stackebrandt & Liesack, 1993), and sequenced using a Sanger DNA sequencer (ABI 3730XL; Applied Biosystems, Foster City, California, United States). The 16S rRNA gene sequence of SW178 was then compared to closely related strains from the GenBank (www.ncbi.nlm.nih.gov/genbank/) and EZBioClound databases (www.ezbiocloud.net/eztaxon) (Kim et al., 2012) as described in previous studies (Ghimire, Wongkuna & Scaria, 2020; Wongkuna et al., 2020). After isolation and identification, the strain was maintained in BHI-M medium and stored with 10% (v/v) dimethyl sulfoxide (DMSO) at −80 °C. The reference strain, *M. torques* ATCC 27756^T^, obtained from the American Type Culture Collection (ATCC), was maintained under the same conditions.

### Genome sequencing and analysis

The whole genome sequencing of strain SW178 was performed using Illumina MiSeq with V3 chemistry. The reads were assembled using Unicycler that built an initial assembly graph from short reads using the de novo assembler SPAdes 3.11.1 (Wick et al., 2017). The quality assessment for the assemblies was performed using QUAST (Gurevich et al., 2013). Genome annotation was performed using Rapid Annotation using Subsystem Technology (RAST) server (Overbeek et al., 2014). Furthermore, species delineation methods were used to prove the novelty of strain SW178. The digital DNA–DNA hybridization (dDDH) was performed between strain 178 and the closet phylogenetic neighbor using Genome-to Genome Distance Calculator (GGDC 2.1) web server (http://ggdc.dsmz.de) (Meier-Kolthoff et al., 2013). Average nucleotide identity (ANI) between strain SW178 and the closely related strains was also calculated using the OrthoANI software (Goris et al., 2007).

### Phylogenetic analysis

To perform phylogenetic analysis, 16S rRNA sequence of strain SW178 was extracted from the annotated data, and blast against EzBioClound to obtain 16S rRNA sequences of closely related strains. Phylogenetic tree of all 16S rRNA sequences was constructed using MEGA7 software (Kumar, Stecher & Tamura, 2016). Multiple sequence alignments were generated using the CLUSTAL-W (Thompson, Higgins & Gibson, 1994). Reconstruction of phylogenetic trees was carried out using Neighbor-Joining method. The distance matrices were generated according to Kimura’s two-parameter model. Bootstrap resampling analysis of 1,000 replicates was performed to estimate the confidence of tree topologies.

### Phenotypic characterization

Colony morphology was determined after 2–3 days of incubation on BHI-M agar plates. Gram-staining was performed using a Gram-Straining kit set (Difco), according to the manufacturer’s instructions. Cell morphology during exponential growth was examined by scanning electron microscopy (SEM). Aerotolerance was examined by incubating the strain for 2 days under aerobic and anaerobic conditions. Growth of strain SW178 at various temperatures including 4, 20, 30, 37, 40 and 55 °C was observed. The optimal pH for growth was determined by adjusting to pH 4–9 with sterile anaerobic solutions of 0.1 M HCl and 0.1 M NaOH. Motility of this microorganism was determined using motility medium with triphenyltetrazolium chloride (TTC) (Shields & Cathcart, 2011). The growth was indicated by the presence of red color due to reduction of TTC after it was absorbed into the bacterial cell wall. The utilization of various substrates and enzyme activities were evaluated with the AN MicroPlate (Biolog, Hayward, California, United States) and API ZYM (bioMérieux, Marcy-l’Étoile, France), according to the manufacturers’ instructions. For cellular fatty acid analysis, strain SW178 was cultured in BHI-M medium at 37 °C for 24 h under anaerobic condition. Cellular fatty acids were obtained from cell biomass and analyzed by GC (7890A; Agilent, Santa Clara, California, United States), according to the manufacturer’s instruction of Microbial Identification System (MIDI) (Sasser, 1990).

### Data availability

The following information was supplied regarding the deposition of DNA sequences:

The 16S rRNA gene sequence was deposited in Genbank under accession number MN133850. The genome sequence data of strain SW178 was available in GenBank BioProject: PRJNA554364.

### New species deposition

The proposed type strains for *Mediterraneibacter catenae* were deposited in the Leibniz Institute DSMZ-German Collection of Microorganisms and Cell Cultures GmbH and the national Culture Collection of Switzerland (CCOS) under number DSM 109242 and CCOS 1886, respectively.

## Results

**Figure 1:**
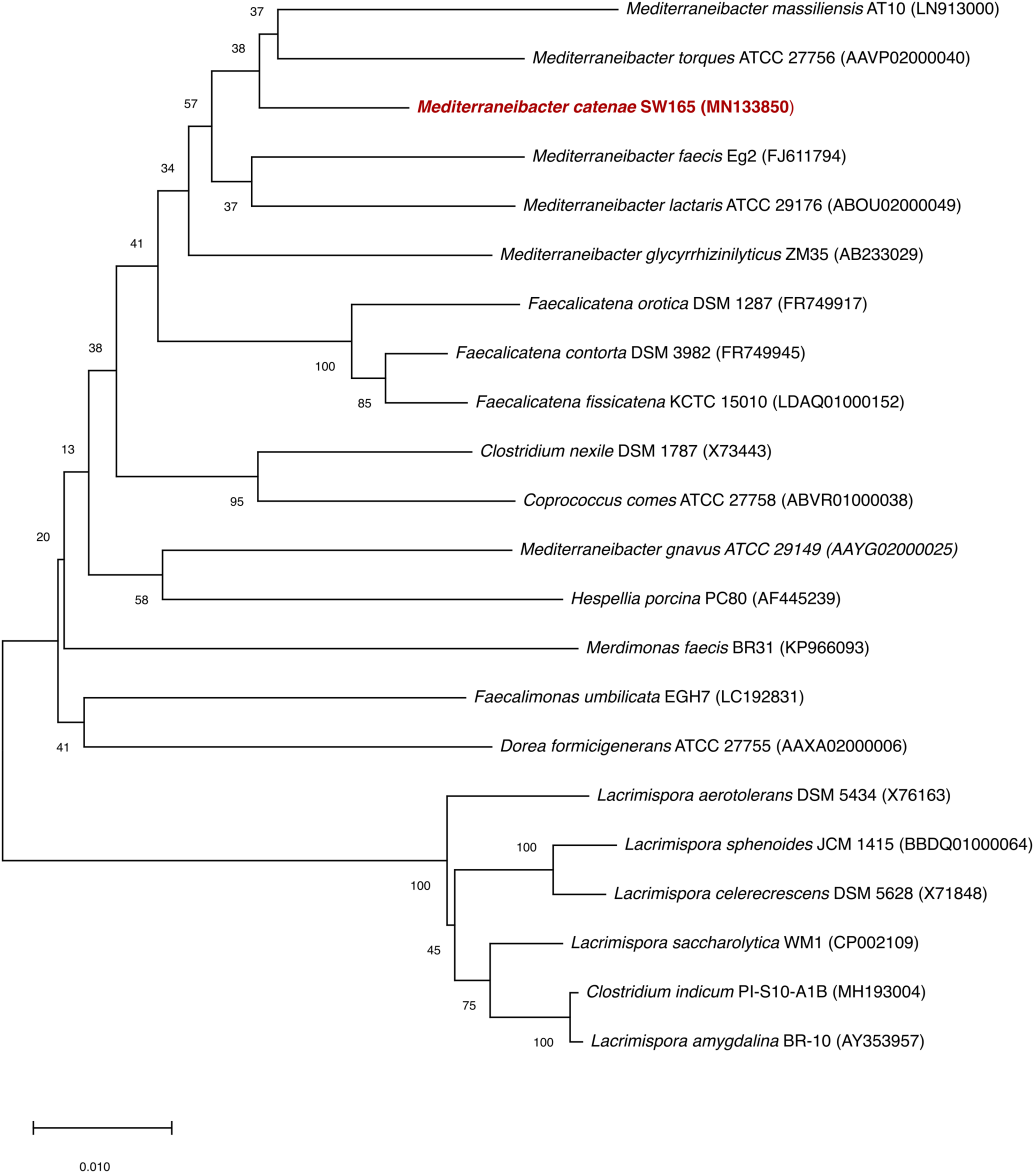
Neighbor joining tree based on 16S rRNA gene sequences of SW178 with its related strains. Genbank accession numbers of the 16S rRNA genes present in parentheses. The sequences were aligned using the CLUSTAL-W before generating phylogenetic trees using MEGA7. The distance matrices were computed using Kimura’s two-parameter modelwith 1,000 bootstrap tests to estimate the confidence of tree topologies. Numbers at nodes are shown as percentages of bootstrap greater than 70%. Bar, 0.01 substitutions per nucleotide position.

Based on 16S rRNA gene sequencing, the closest relatives of SW178 were *M. torques* ATCC 27756^T^ with 96.94% similarity, followed by *M. glycryrrhizinilyticus* ZM35^T^ (96.70%), *M. massiliensis* Marseille-P2086^T^ (96.54%), *M. faecis* Eg2^T^ (96.31%) and *M. lactaris* ATCC 29176^T^ (95.81%). The results of phylogenetic analysis revealed that strain SW178 was clustered within the family *Lachnospiraceae*, indicating that the strain represents a novel member of this family (Fig. 1).

**Figure 2:**
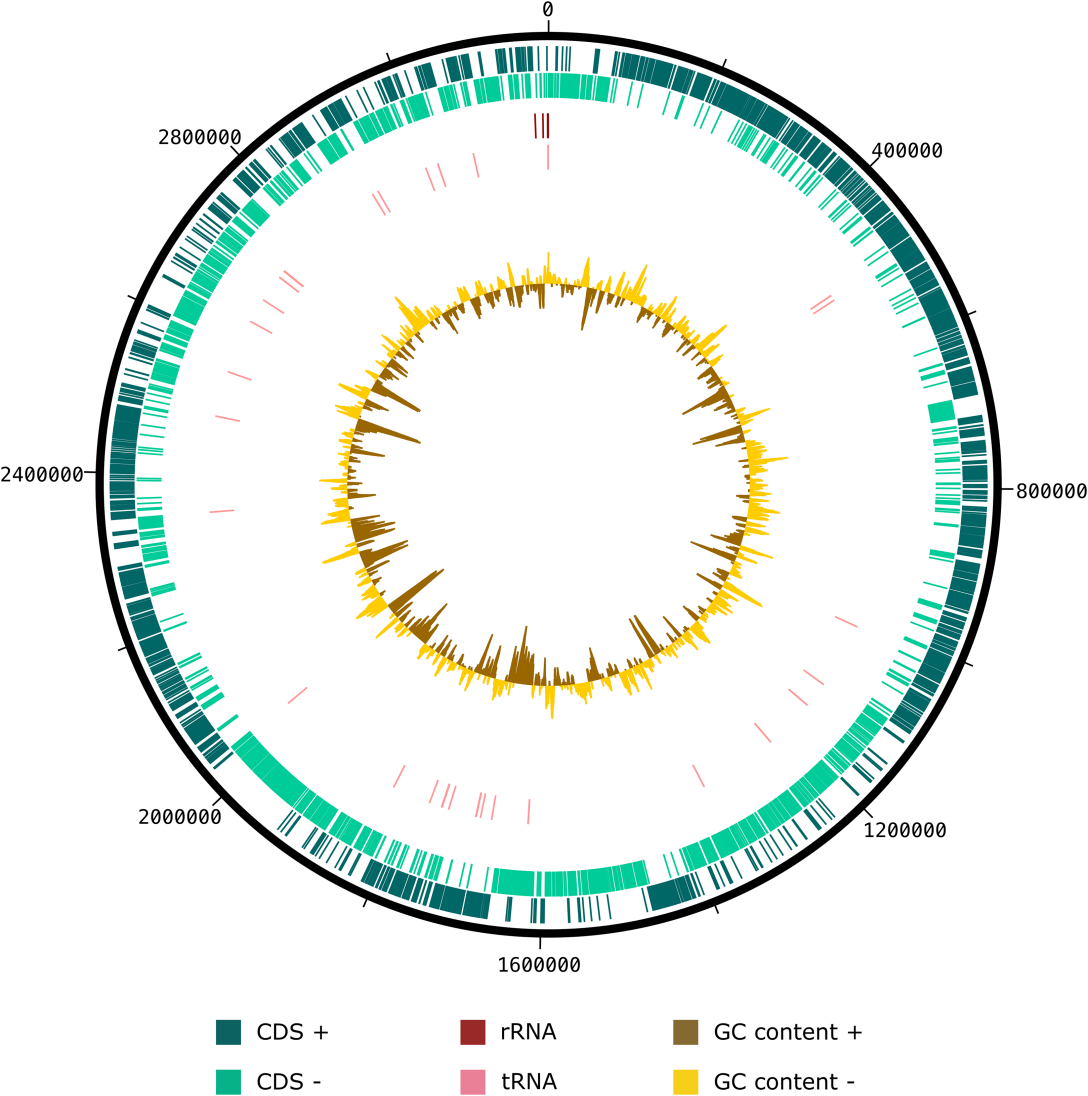
Graphical circular map of SW178 genome. The assembled genome of the strain was used to generate a circular graph using DNAplotter. From outside to the center: coding sequences on the forward strand (CDS +), coding sequences on the reverse strand (CDS −), tRNAs, rRNAs, GC content and GD skew.

**Figure 3:**
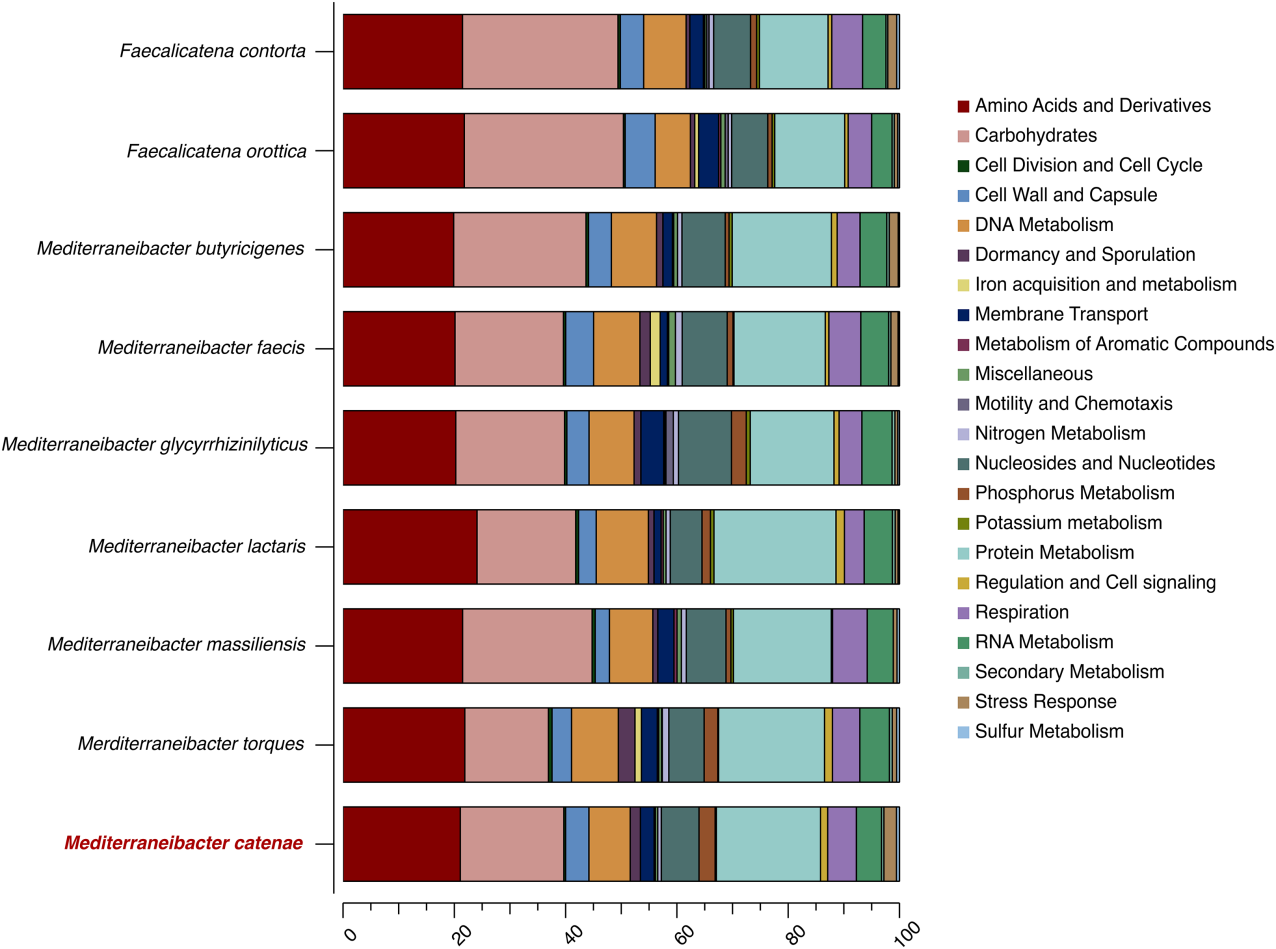
Distribution of functional features of predicted coding sequences of SW178 and its neighbors. The functional features were predicted based on the clusters of orthologous groups. Heatmap was generated from genome annotation of individual species by RAST using Explicet software.

**Figure 4:**
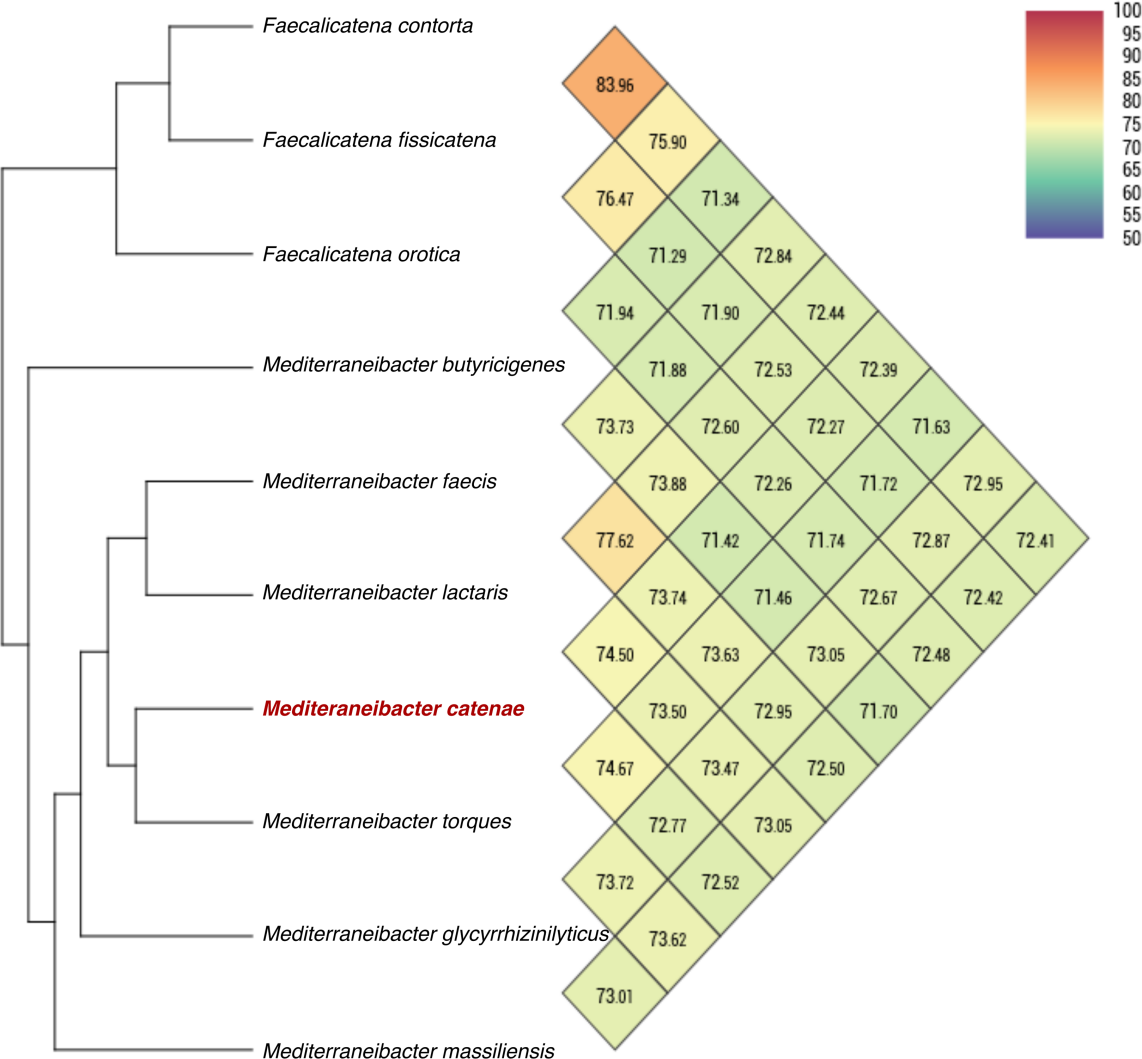
Average nucleotide identity comparison of SW178 and closely related strains. Genome sequences of the strain and related taxa with valid taxonomy were used for calculation of average nucleotide identity (ANI). Heatmap represents OrthoANI values generated using OAT software.

The draft genome sequence of strain SW178 has a total length of 3.18 Mbp and 46.9 mol% of G+C content (Fig. 2). In the genome of strain SW178, 3,206 genes were coding sequences, while 56 genes were RNAs (48 tRNAs and eight rRNAs). Total 1,110 genes were classified in functional categories (COGs) (Table S1). The major functional categories on the genome of strain SW178 included amino acids and derivatives, carbohydrates, and protein metabolism, while the absent categories were cell division and cell cycle, iron acquisition and metabolism, metabolism of aromatic compounds, motility and chemotaxis, and potassium metabolism (Fig. 3). Strain SW178 showed the most similar COGs pattern to *M. torques*, which is the closest strain based on phylogenetic analysis. Additionally, overall patterns of COGs were similar among members of the genus *Mediterraneibacter*, whereas they were distinct from the genus *Faecalicatena*, which are also chain-shaped microorganisms isolated from feces belonging to the family of *Lachnospiraceae*. In particular, the function involved in protein metabolism showed a larger ratio in *Mediterraneibacter* compared to *Faecalicatena*. Conversely, the portion of function involved in carbohydrates found in *Faecalicatena* species was greater than that in *Mediterraneibacter*. The average nucleotide identity (ANI) between strain SW178 and the closely related strains were 71.63–74.70% (Fig. 4). The ANI values were significantly less than the proposed ANI cutoff of 95–96 % (Kim et al., 2014), indicating that strain SW178 is a novel species within the genus *Mediterraneibacter*. Additionally, dDDH value between strain SW178 and *M. torques* ATCC 27756^T^, the closest valid phylogenetic taxa were 18.2% ± 3.4% with 4.97% difference in G+C content, suggesting distinct species.

**Figure 5:**
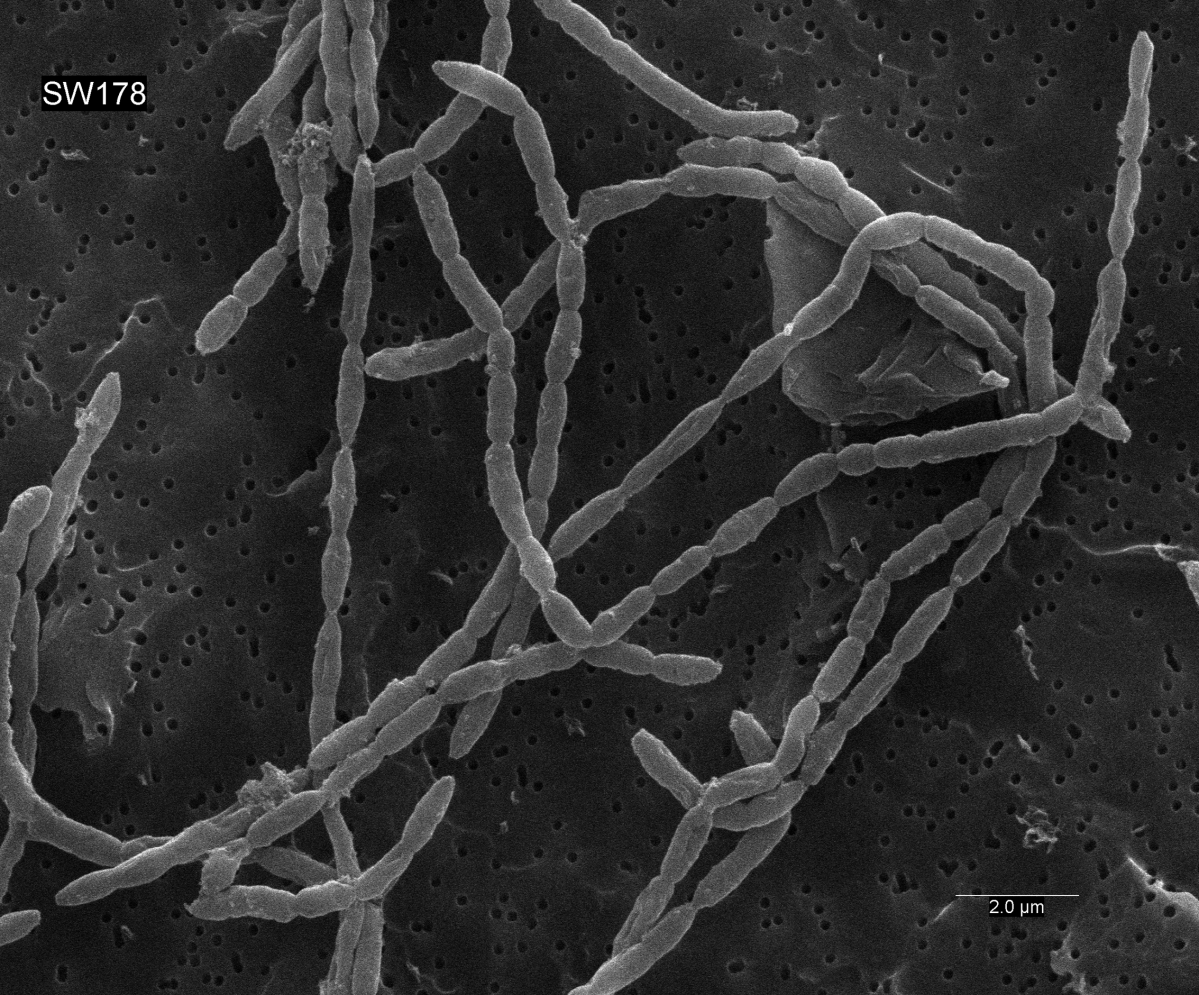
Scanning electron micrograph of strain SW178. Cells were anaerobically cultured for 24 h at 37 °C in BHI-M medium before image using scanning electron microscopes. Bar, 2 μm.

Cells of strain SW178 were Gram-positive coccobacilli with 0.5–1.0 µm growing in chain (Fig. 5). Colonies on BHI-M agar were white, raised, and entire edges with 0.1–0.3 cm in diameter. Strain SW178 grew between 37 and 45 °C with the optimum growth at 45 °C. The growth was observed at pH between 6.5 to 7.5 with the optimum pH at 7.5. The strain grew only under anaerobic condition, indicating an obligately anaerobic bacterium. Based on the results obtained in Biolog tests, strain SW178 utilized dextrin, L-fucose, D-galacturonic, α-D-glucose, L-rhamnose and D-sorbitol. API ZYM results showed that the strain exhibited positive detection of esterase (C4), esterase lipase (C8), β-galactosidase, α-glucosidase and β-glucosidase. (Table 1). The predominant cellular fatty acids of strain SW178 included C_14 : 0_ (28.71%), C_16 : 0_ (19.55%) and C_16 : 0_ dimethyl acetal (DMA) (12.55%), which were different from the reference strain (Table 2).

**Table 1:** Characteristics of strain SW178 and its relatives.

Characteristic	SW178	ATCC 27756^T^	KCTC 15684^T^	KCTC 5760^T^	KCTC 5757^T^	DSM 100837^T^	ATCC 29176^T^
Isolation source	Chicken cecum	Human feces	Human feces	Human feces	Human feces	Human feces	Human feces
Gram stain	+	+	+	+	+	+	+
Growth at 45 °C	+	+	−	−	−	+	−
pH 5	−	−	−	−	−	−	+
Motility	non-motile	non-motile	non-motile	non-motile	non-motile	non-motile	non-motile
**Carbon source (BIOLOG AN)**							
Adonitol	−	−	−	−	NA	NA	NA
Amygdalin	+	+	NA	NA	NA	NA	−
Dextrin	−	+	+	w	−	NA	NA
D-Fructose	+	−	+	−	NA	+	+
α-D-Glucose	−	+	+	+	+	+	+
Glycerol	−	−	+	−	NA	NA	NA
D-Mannose	−	+	+	+	−	NA	w
Palatinose	+	−	NA	NA	NA	+	NA
L-Rhamnose	+	+	+	−	−	NA	−
α-Ketobutyric Acid	+	+	+	−	NA	−	NA
L-Serine	−	−	+	−	NA	NA	NA
Thymidine	−	−	+	−	NA	NA	NA
**Enzyme activity (API ZYM)**							
Alkaline phosphatase	−	+	+	+	NA	NA	NA
Valine arylamidase	−	−	NA	NA	NA	NA	NA
Trypsin	−	−	NA	NA	NA	NA	NA
α-galactosidase	−	−	+	−	NA	NA	NA
β-galactosidase	+	−	−	+	NA	NA	NA
β-glucuronidase	−	−	NA	NA	NA	NA	NA
α-glucosidase	+	−	NA	NA	NA	NA	NA
β-glucosidase	+	−	NA	NA	NA	NA	NA
DNA G+C content (mol%)	49.6	40	44.1	45.7–4.2	43.4	42.4	445

**Notes:**

+, positive; −, negative; w, weak positive; NA, not available.

Strains: 1, SW178; 2, *Mediterraneibacter torques* ATCC 27756^T^; 3, *Mediterraneibacter butyricigenes* KCTC 15684^T^; 4, *Mediterraneibacter glycyrrhiziilyticus* KCTC 5760T; 5, *Mediterraneibacter faecis* KCTC 5757^T^; 6, *Mediterraneibacter massiliensis* DSM 100837^T^; 7, *Mediterraneibacter lactaris* ATCC 29176^T^. Cells were anaerobically cultured in BHIM at 37 °C for 2 days.

**Table 2:** Cellular fatty acid contents (%) of strain SW178 and its closely related strains.

Fatty acid composition	SW178	ATCC 27756^T^	KCTC 15684^T^	KCTC 5760^T^	KCTC 5757^T^	DSM 100837^T^
**Straight chain**						
C_12 : 0_	1.31	2.9	1.8	1.9	NA	–
C_14 : 0_	28.71	7.98	21.2	31.3	10	2.0
C_16 : 0_	19.55	23.58	16.4	12.4	27.7	54.0
C_16 : 0_ ALDE	3.35	2.46	1.4	2.7	NA	NA
C_18 : 0_	1.15	9.96	3.5	2.0	2.9	9.0
**Demethylacetal (DMA)**						
C_14 : 0_ DMA	7.82	0.78	5.0	13.1	NA	NA
C_16 : 0_ DMA	12.55	9.66	5.1	7.9	NA	NA
C_18 : 0_ DMA	1.73	3.42	3.0	2.2	NA	NA
C_16 : 1_ω7*c* DMA	2.41	–	–	–	NA	NA
C_18 : 1_ω7*c* DMA	7.9	–	–	–	NA	NA
**Unsaturated**						
C_13 : 1_ at 12–13	4.26	0.38	–	–	NA	
C_16 : 1_ ω7*c*	1.42	10.26	–	–	NA	–
C_16 : 1_ ω9*c*	–	6.15	4.3	–	2.5	–
C_18 : 1_ ω7*c*	1.95	3.57	–	–	NA	2.0
C_18 : 1_ ω9*c*	0.78	16.71	7.1	5.5	3.1	30.0
Summed feature 1*	4.26	0.38	2.3	7.2	NA	NA

**Notes:**

* Summed features are fatty acids that could not be separated using the MIDI System. Summed feature 1 contains C_13 : 1_ and/or C_14 : 0_ aldehyde.

Strains: 1, SW178; 2, *Mediterraneibacter torques* ATCC 27756^T^; 3, *Mediterraneibacter butyricigenes* KCTC 15684^T^; 4, *Mediterraneibacter glycyrrhizinilyticus* KCTC 5760^T^; 5, *Mediterraneibacter faecis* KCTC 5757^T^; 6, *Mediterraneibacter massiliensis* DSM 100837^T^. Strains were anaerobically cultured for 2 days in modified brain heart infusion (BHI-M). Fatty acids comprising less than 1% of the total fatty acids in all strains were not shown.

## Discussion

Metagenomics using next generation sequencing (NGS) is the primary strategy for the study of gut microbiota (Dhakan et al., 2019). It has revealed the compositional information of microbial community residing in the gut, in which a large number of the detected bacteria are uncultured (Lagier et al., 2016). To manage this issue, culture-dependent methods known as “culturomics” have been developed to characterize and identify previously unculturable bacteria, allowing additional known members of gut microbiota (Kaeberlein, Lewis & Epstein, 2002; Lagier et al., 2015). Further, the pure culture of bacteria is crucial to emphasize their functions, and interactions in health and diseases for supportive and therapeutic approaches such as the development of new-generation probiotics (Goodman et al., 2011; Rettedal, Gumpert & Sommer, 2014). In this study, we employed the culture-based isolation to recover the previously uncultured bacterium SW178 from the cecum content of feral chickens and describe its characteristics using taxono-genomics methods as a new bacterial species.

Taxono-genomics becomes the gold standard to determine the novelty of a prokaryotic organism (Fournier & Drancourt, 2015). Homology of the 16S rRNA gene sequence is primarily used to differentiate at taxonomic levels with the cut-off point of 98.65% similarity. In addition, the threshold values of 95-96% ANI and 70% DDH are generally recommended for prokaryotic species demarcation based on genome sequence similarity (Goris et al., 2007; Kim et al., 2014; Richter & Rossello-Mora, 2009). The analysis based on 16S rRNA showed that strain SW178 clustered with *Mediterraneibacter* members with 96.94% similarity to *M. torques* ATCC 27756^T^, the closest related species (Fig. 1). The genome sequence comparison between stains SW178 and other relative species revealed the value range of 71.63–74.67% ANI (Fig. 2). These values were less than cut-off points for bacterial species delineation, suggesting strain SW178 as a novel member of *Mediterraneibacter* genus in the family *Lachnospiraceae*. The members of *Mediterraneibacter* were currently reclassified from the genus *Ruminococccus* which are Gram-positive, non-motile and strictly anaerobic bacteria. The range of G+C content is 42–46 mol% (Togo et al., 2018). In addition to 16S rRNA- based analysis, whole genome comparison was performed to determine taxonomic delineation which showed distance between the strain and its closely related strains (Figs. 3 and 4). Strain SW178 also exhibited distinct phenotypic properties to its closest valid strain, including carbon source utilization, enzymatic activity, and cellular fatty acid components (Tables 1 and 2).

On the basis of the genotypic and phenotypic features of strains SW178, this organism identified herein should be described as a novel species of the genus *Mediterraneibacter*. We assigned strain SW178 as the type strain of a novel species, for which the name *Mediterraneibacter catenae* is proposed.

## Conclusion

Here we described *Mediterraneibacter catenae* sp. nov. (ca.te’nae. L. Pl. n. *catenae*, chains referring to cells growing in chains).

Cells are strictly anaerobic, Gram-stain-positive and non-motile. The average size of each cell is 0.5–1.0 µm in oval shaped growing in chains. Colonies are visible on BHI-M agar after 2 days and are approximately 0.1–0.3 cm in diameter, white, raised and circular with entire edges. The microorganism exhibits optimal growth in BHI-M medium at 45 °C and pH 7.5. The strain utilizes dextrin, L-fucose, D-galacturonic, α-D-glucose, L-rhamnose and D-sorbitol. The primary cellular fatty acids are C_14 : 0_ , C_16 : 0_ and C_16 : 0_ DMA. The genome of this strain is 3.18 Mbp with 46.9 mol% of G+C content. This strain was isolated from the cecum of feral chickens. The type strain is SW178 (= DSM 109242^T^ = CCOS 1886^T^)

## Supplemental Information

10.7717/peerj.11050/supp-1 Supplemental Information 1 Functional categories (COGs) from genome of strain SW178.

## Additional Information and Declarations

### Data Availability

The following information was supplied regarding data availability: The 16S rRNA gene sequence is available at Genbank: MN133850. The genome sequence data of strain SW178 is also available at GenBank: PRJNA554364. The proposed type strains for *Mediterraneibacter catenae* were deposited in the Leibniz Institute DSMZ-German Collection of Microorganisms and Cell Cultures GmbH and the national Culture Collection of Switzerland (CCOS) under number DSM 109242 and CCOS 1886, respectively.

### New Species Registration

The following information was supplied regarding the registration of a newly described species: The proposed type strains for Mediterraneibacter catenae were deposited in the Leibniz Institute DSMZ (https://www.dsmz.de/collection)-German Collection of Microorganisms and Cell Cultures GmbH and the national Culture Collection of Switzerland (CCOS, https://www.ccos.ch/) under number DSM 109242 and CCOS 1886, respectively.
